# The Biochemical Literacy Framework: Inviting pedagogical innovation in higher education

**DOI:** 10.1002/2211-5463.12938

**Published:** 2020-08-18

**Authors:** Danielle L. Evans, Sarah G. Bailey, Alfred E. Thumser, Sarah L. Trinder, Naomi E. Winstone, Ian G. Bailey

**Affiliations:** ^1^ Department of Biochemical Sciences University of Surrey Guildford UK; ^2^ Department of Higher Education University of Surrey Guildford UK

**Keywords:** biochemistry, curriculum design, higher education, pedagogy, scientific literacy

## Abstract

When developing meaningful curricula, institutions must engage with the desired disciplinary attributes of their graduates. Successfully employed in several areas, including psychology and chemistry, disciplinary literacies provide structure for the development of core competencies‐pursuing progressive education. To this end, we have sought to develop a comprehensive blueprint of a graduate biochemist, providing detailed insight into the development of skills in the context of disciplinary knowledge. The Biochemical Literacy Framework (BCLF) aspires to encourage innovative course design in both the biochemical field and beyond through stimulating discussion among individuals developing undergraduate biochemistry degree courses based on pedagogical best practice. Here, we examine the concept of biochemical literacy aiming to start answering the question: What must individuals do and know to approach and transform ideas in the context of the biochemical sciences? The BCLF began with the guidance published by relevant learned societies – including the Royal Society of Biology, the Biochemical Society, the American Society for Biochemistry and Molecular Biology and the Quality Assurance Agency, before considering relevant pedagogical literature. We propose that biochemical literacy is comprised of seven key skills: critical thinking, self‐management, communication, information literacy, visual literacy, practical skills and content knowledge. Together, these form a dynamic, highly interconnected and interrelated meta‐literacy supporting the use of evidence‐based, robust learning techniques. The BCLF is intended to form the foundation for discussion between colleagues, in addition to forming the groundwork for both pragmatic and exploratory future studies into facilitating and further defining biochemical literacy.

## Graphical abstract

What makes a biochemist? Here, we introduce the Biochemical Literacy Framework (BCLF) comprised seven key skills: critical thinking, self‐management, communication, information literacy, visual literacy, practical skills and content knowledge. This BCLF is intended to facilitate further consultation and discussion towards defining the context and qualities of a biochemist.

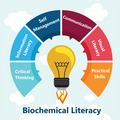

**Abbreviations**
ASBMB: American Society for Biochemistry and Molecular Biology
BCLF: Biochemical Literacy Framework
BSCS: Biological Sciences Curriculum Studies
NUS: National Union of Students
QAA: Quality Assurance Agency
RSB: Royal Society of Biology

The modern employment market means that graduates must be freethinking and adaptable [1], preparing for a career filled with challenges and change. To develop meaningful curricula, it is essential that institutions engage with the desired disciplinary attributes of their graduates. An aim is to develop individuals who are able to adapt through questioning and investigation to develop a career about which they are passionate and which they enjoy. Whilst biochemistry graduates have the potential to be biochemists, their development is not restricted to one career path, with biochemists fulfilling roles from technical and research to administrative and sales [2].

For undergraduate biochemistry students, biochemistry is simply the context in which key skills and attributes are learnt and developed. There is little concrete restriction on the topics taught in an undergraduate biochemistry degree – paving the way for institutions to play to their strengths and produce engaging teaching around ‘hot topics’ at the forefront of research. However, in contrast to some current teaching practice at undergraduate level, skills should be taught in the context of disciplinary content knowledge [3, 4]. Skills should be the focus, facilitating understanding including the development of connections between topics creating an independent, adaptable learner [5, 6, 7].

Teaching skills in the context of disciplinary knowledge could support evidence‐based design for learning within programmes, focusing on the interconnections of biochemical knowledge, fostering lifelong learning skills and developing confident curious open‐minded biochemists ready to integrate and participate in society. Individuals who are highly literate in the biochemical sciences can draw upon their skill set to apply themselves to new challenges as they desire with little constraint.

High‐quality science education sustains a dynamic scientific community able to address global problems, and encourages an increased scientific literacy in the general population [8, 9]. With these goals in mind, it is imperative that teaching and assessment strategies at all levels are approached with the same inquiry‐driven, evidence‐based approach as our scientific research, despite the challenges this may involve.

Bybee [10] and Shamos [11] proposed multiple levels of scientific literacy, increasing in complexity. The four levels suggested by Bybee [10] and the Biological Sciences Curriculum Studies (BSCS) [12] (Table 1) were one of the key theoretical frameworks underpinning the definition of chemical literacy [13] and thus inform our own approach to defining biochemical literacy.

**Table 1:** The scale of scientific literacy, suggested by [10] and [12], as adapted from [13].

Scientific literacy categories	Definitions
Scientific illiteracy	Students who cannot relate to, or respond to a reasonable question about science. They do not have the vocabulary, concepts, contexts or cognitive capacity to identify the question as scientific
Nominal scientific literacy	Students recognise a concept as related to science, but the level of understanding clearly indicates misconceptions
Functional scientific literacy	Students can describe a concept correctly, but have a limited understanding of it
Conceptual scientific literacy	Students develop some understanding of the major conceptual schemes of a discipline and relate those schemes to their general understanding of science. Procedural abilities and understanding of the processes of scientific inquiry and technological design are also included in this level of literacy
Multidimensional scientific literacy	This perspective of scientific literacy incorporates an understanding of science that extends beyond the concepts of scientific disciplines and procedures of scientific investigation. It includes philosophical, historical and social dimensions of science and technology. Here, students develop some understanding and appreciation of science and technology regarding its relationship to their daily lives. More specifically, they begin to make connections within scientific disciplines, and between science, technology and the larger issues challenging society

## Existing disciplinary literacy frameworks

**Fig. 1:**
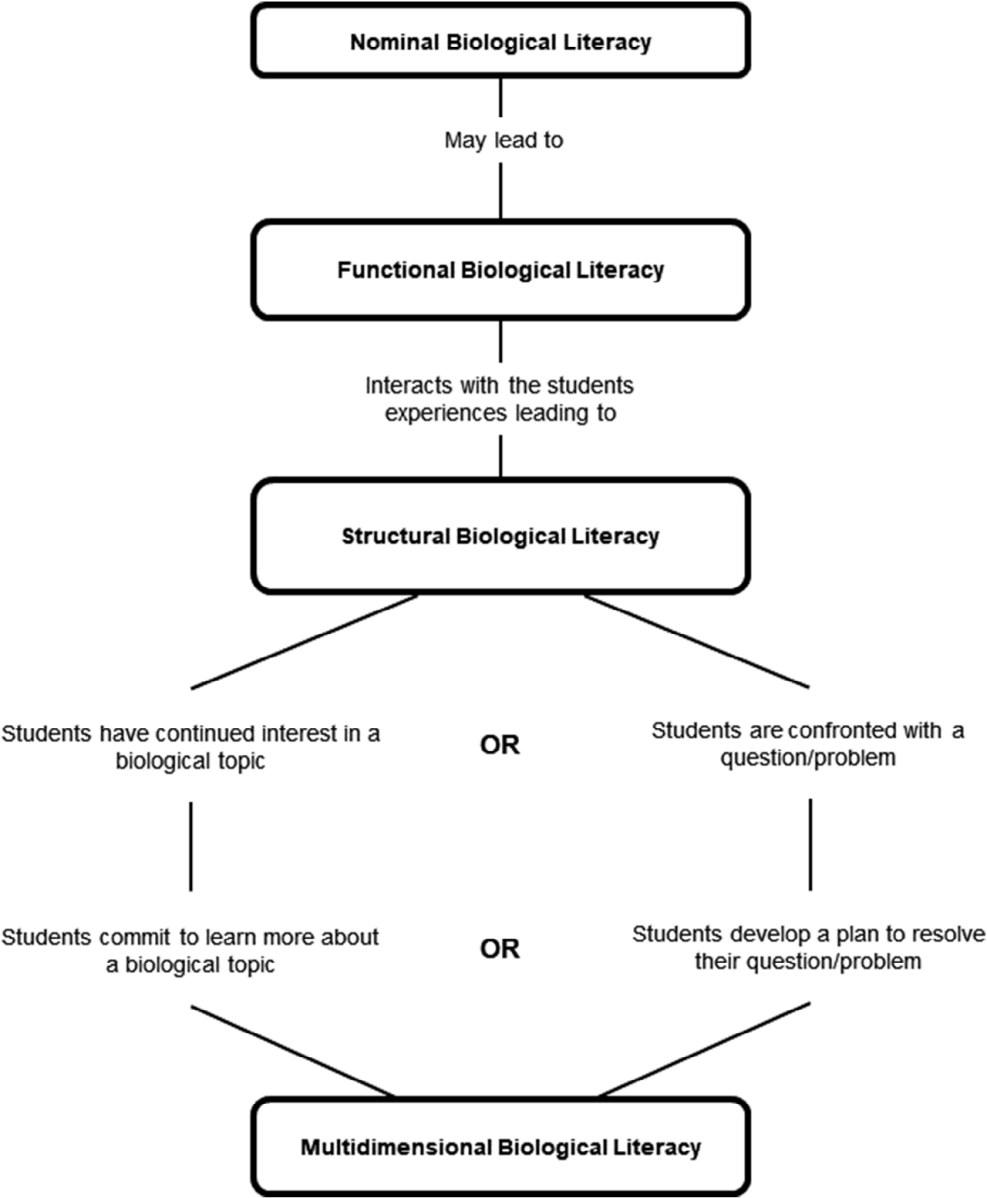
Biological literacy model. The model of biological literacy developed by the Biological Sciences Curriculum Studies, adapted from [12].

Other disciplines have undertaken more advanced investigations into both best pedagogic practices, and literacy within their speciality – in particular, we have referred to existing research in chemistry, biology and psychology [14, 15, 16, 17, 18, 19] – to inform the construction of the biochemical literacy framework (BCLF). For example, the development of biological literacy by the BSCS [12] produced the model of biological literacy, which shows the interaction between the four levels of scientific literacy given in Table 1. This model is shown in Fig. 1 and directed the format of the BCLF wherein interconnections were explicitly shown.

In these disciplinary frameworks, scientific literacy has been positioned as being underpinned by disciplinary literacy [13]. It is important to note that attainment of high scientific literacy does not mean an individual always has high disciplinary literacy. For example, a geologist can be scientifically literate, but not biochemically literate, and vice versa. Scientific literacy crosses the disciplines, whereas disciplinary literacy does not. Scientific literacy and the importance of scientific literacy to the general population are discussed later.

When examining these literacies, we learnt that there is unlikely to be a ‘one‐size‐fits‐all’ approach with such a diverse and multidisciplinary topic as biochemistry, with both chemistry and biology remaining active areas of pedagogic discussion [20, 21, 22] and innovation [23, 24, 25, 26]. This approach aligns with the Quality Assurance Agency (QAA) and Royal Society of Biology (RSB) guidance – with neither aiming to provide a prescriptive curriculum, but encouraging creativity and innovation [1, 27].

There are several existing concept inventories for the biochemical sciences, most notably the work of Loertscher *et al*. [28] on identifying the threshold concepts for biochemistry. However, no consensus on the key skills underpinning the biochemical sciences is readily available to educators. To this end, we have sought to develop a comprehensive blueprint of a graduate biochemist, providing detailed insight into the development of skills in the context of disciplinary knowledge. This is intended as a foundation document, with an invitation to colleagues to further develop these, in order that biochemistry curricula are developed for high quality, capable and independent graduates.

The overall aim of when formulating the BCLF was to construct a clear framework of the capabilities composing ‘Biochemical Literacy’. We aimed to achieve this through two objectives: firstly, collating the key capabilities embedded within guidance published by learned societies relating to the development of undergraduate Biochemical Sciences programmes, and identifying themes within and across documentation; and secondly, identifying literature relevant to each theme, utilising systematic literature searching techniques in order to provide clarity and depth to the framework.

The BCLF could prove invaluable in assisting the production of higher quality courses by initiating discussion among those developing biochemical degree courses, in particular regarding pedagogic best practices as the foundation of the curriculum. There are several pedagogical approaches implicitly supported by the framework due to their alignment with the idea of teaching skills in the context of content knowledge [29, 30]. These move teaching methods towards student‐centred learning – actively involving students in their education and facilitating lifelong learning practices [6].

## Materials and methods

The data for this study consisted of guidance documents published by the scholarly, statutory and specialist organisations: the QAA, RSB, the Biochemical Society, Advance HE (previously known as the Higher Education Academy) and the American Society for Biochemistry and Molecular Biology (ASBMB). These represent the main sources of reference for the creation and content of undergraduate biochemistry courses in UK Higher Education Institutions and are all available in the public domain. Each of these documents has undergone development and/or validation processes as detailed in Table 2.

**Table 2:** The development and/or validation processes of the undergraduate Biochemistry Curriculum guidance documentation, collated from each individual guidance source – citations embedded for clarity.

Guidance document	Development and/or validation processes
RSB accreditation	Initial (2010) input from: universities, business and industry, government, learned societies, research councils, funding bodies and sector skills councils. Two‐year consultation period, including a survey of undergraduate, postgraduate and recent graduate students of the life sciences. As of 2018, accreditation conference attendees input into the accreditation quinquennial review via round table discussions [69, 129]
QAA parts and chapters	Consultation with higher education providers; their representative bodies; the NUS; professional, statutory and regulatory bodies; and other interested parties [130]
QAA subject benchmark statement: biosciences	Produced by a group of subject specialists drawn from, and acting on behalf of, the subject community. This then goes through a full consultation with the wider academic community and stakeholder groups, all facilitated by the QAA [27]
ASBMB	Five‐phase project involving disciplinary experts and students, in addition to high school, college and university educators. This process was undertaken by Loertscher *et al*. funded by the National Science Foundation (NSF) and is detailed in [28]

Additional material was identified when referring to the bibliographies of guidance provided by relevant organisations, before finally additional scholarly guidance were identified through a series of searches utilising the online databases PubMed, ERIC and Google Scholar. Only literature written in the English Language where the full text was available, or obtainable within the study time frame through interlibrary requests were included.

Thematic analysis provided both the overarching concepts, and much of the detail present in the BCLF. The data were coded inductively, identifying key words common to all documentation beginning with the UK QAA subject benchmark statement for the biosciences [27] then moving to the more disciplinary‐specific guidance. The common key terms were grouped, using a concept mapping [31, 32, 33] approach to develop succinct overviews of areas derived from multiple sources of literature. A ‘bigger picture’ order was developed by considering overlapping and associated elements across multiple concept maps. These thereafter went through many stages of reduction aiming for clarity of communication without compromising quality before producing the final framework. Comprehensibility was considered at every stage, therefore informing subsequent stages. This facilitated the identification and organisation of the forming seven skill areas underpinning biochemical literacy.

## Results and Discussion

Building on our interpretation of literacy, we begin to explore it in the context of the biochemical discipline. Defining the skills and foundational knowledge underpinning disciplinary literacy is complex and multifaceted [18] due to the complex nature of skills and knowledge with their many interconnections. The disciplinary literate individual possesses skills for lifelong learning in their field of study and their literacy comprises multiple core interacting skills, which we have grouped as following: [34, 35]

- Critical thinking
- Communication
- Self‐management
- Information literacy
- Visual literacy
- Practical skills
- Content knowledge

**Fig. 2:**
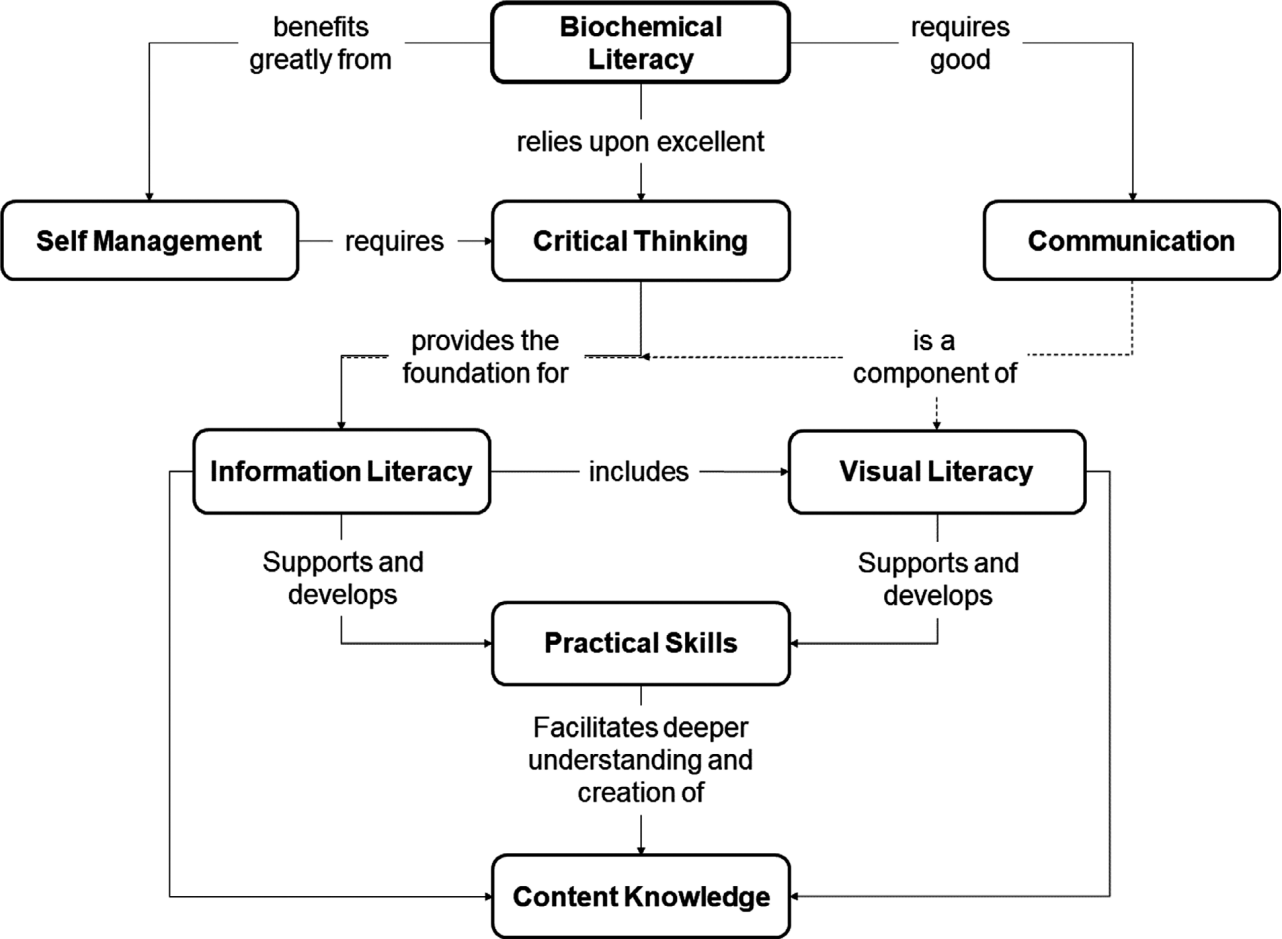
Biochemical literacy. A concept map illustrating the core‐interacting skills forming the foundation of biochemical literacy.

The grouping of these skills is almost immaterial beyond assisting clarity of communication – what matters is how they interact, that is their connections together with the discussion they elicit when designing and planning a course of learning. These connections are illustrated in Fig. 2 following a concept map format. Interactions between these core skills are keys because progression in one can permit new perspectives, transforming understanding. Without these skills, limits upon learning and development are imposed upon the individual.

### Critical thinking: a contextual, self‐improving process

Rigorous, well‐evidenced and inquiry‐based evaluation is fundamental to ‘thinking like a scientist’ [36, 37, 38]. Undergraduates are encouraged to question everything from information, to conclusions and points of view [39, 40, 41]. An essential part of this is the determination of the scientific integrity of information, by ensuring that robust and unbiased methodology is present, both in the work of others and in their own [35, 42, 43].

This self‐improving cycle of ‘scientific thinking’ is supported by the general principles of ‘critical thinking’ [42, 44, 45]. Whilst critical thinkers are found outside the sciences, the inwardly evaluative nature of scientific enquiry means that the development of critical thinking skills is well grounded in STEM curricula [36, 39].

Critical thinking has been discussed by several within the Scholarship of Teaching and Learning in relation to the biosciences [46, 47]. However, we find the most complete, clear and robust definition is offered by Scheffer *et al*. [48] who examined the concept using the Delphi technique, thus generating both discussion and judgement on the topic of critical thinking from multiple experts, which is summarised in Table 3. The differences between critical thinking in nursing, or any other discipline, and biochemistry are fundamentally the context in which they are learnt and developed. For example, a biochemist may develop their critical thinking skills when engaging in a teaching laboratory exercise; perseverance for example is needed when facing challenges, reflection when discovering a result which does not fit with their previous knowledge of a concept, and so on.

**Table 3:** The skills composing critical thinking and their definitions, adapted from Scheffer *et al*. [48].

Skills	Definition
Habits of the mind	
Confidence	Assurance of one's reasoning abilities
Creativity	Intellectual inventiveness used to generate, discover or restructure ideas; imagining alternatives
Flexibility	Capacity to adapt, accommodate, modify or change thoughts, ideas and behaviours
Inquisitiveness	An eagerness to know by seeking knowledge and understanding through observation and thoughtful questioning in order to explore possibilities and alternatives
Intellectual integrity	Seeking the truth through sincere, honest processes, even if the results are contrary to one’s assumptions and beliefs
Intuition	Insightful sense of knowing without conscious use of reason
Open mindedness	A view point characterised by being receptive to divergent rules and sensitive to one’s biases
Perseverance	Pursuit of a course with determination to overcome obstacles
Reflection	Contemplation upon a subject, specially one’s assumptions, and thinking for the purposes of deeper understanding and self‐evaluation
Skills	
Analysing	Separating or breaking a whole into parts to discover their nature, function and relationships
Applying standards	Separating or breaking a whole into parts to discover their nature, function and relationships
Discriminating	Recognising differences and similarities among things or situations and distinguishing carefully as to category or rank
Information seeking	Searching for evidence, facts or knowledge by identifying relevant sources and gathering objective, subjective, historical and current data from these sources
Logical reasoning	Drawing inferences or conclusions that are supported in or justified by evidence
Predicting	Envisioning a plan and its consequences
Transforming knowledge	Changing or converting the condition, nature, form or function of concepts among contexts

To a biochemist, critical thinking could be considered as an independent, controlled, self‐monitored and self‐improving process forming the foundation for all other skills [6, 39, 44, 48]. Critical thinking itself is highly contextual [49], and a simple definition may be considered as:

Determining connections to make evidenced‐based conclusions whilst utilizing evaluation and amalgamation of information [35, 50]

**Fig. 3:**
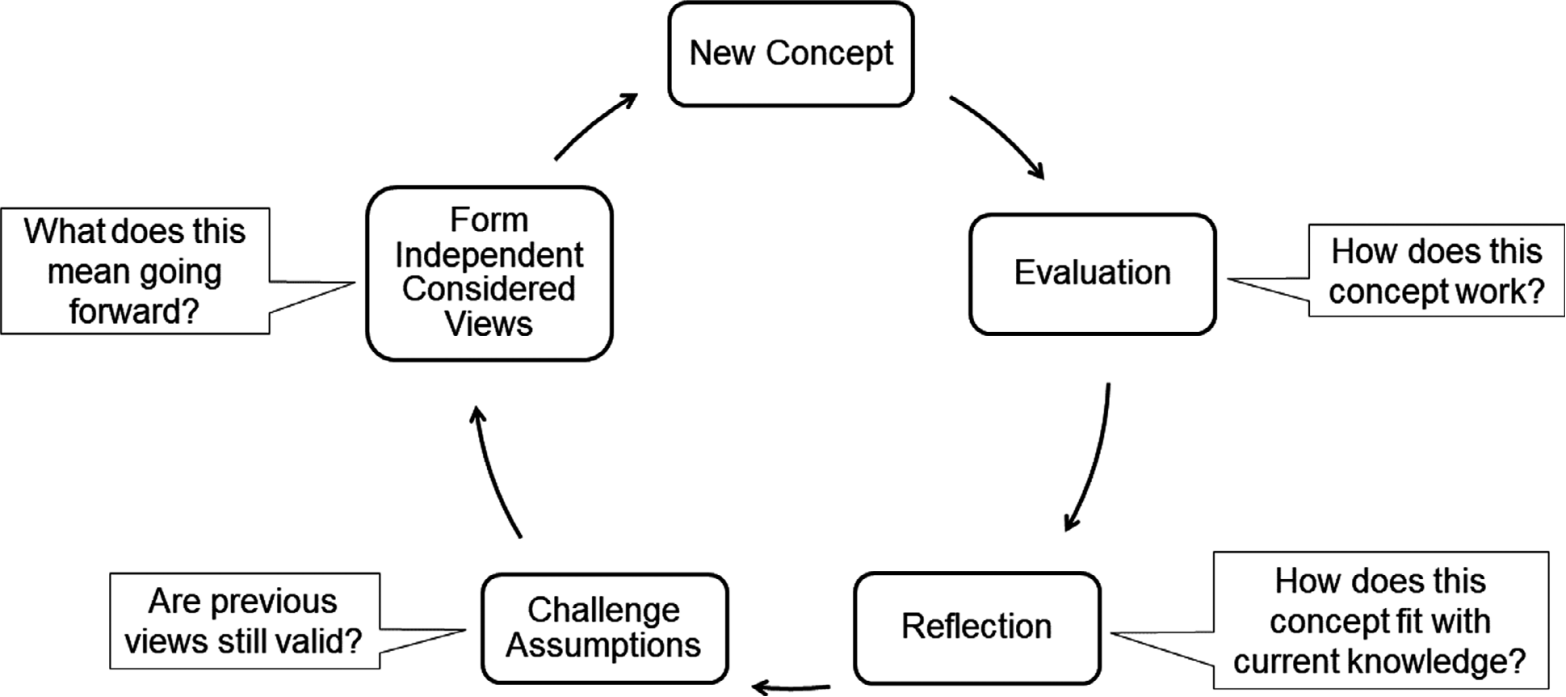
The concept integration cycle. This is a simplistic representation of the critical integration of new or unfamiliar concepts into current understanding through evaluation, reflection, assumption challenge and the formation of independent considered views.

We have illustrated this in Fig. 3, which shows a skeletal overview of related principles. This may provide a useful tool in developing students' critical thinking skills, which they may then use to address information from all sources.

Critical thinking, like many other skills, cannot be learnt easily and must be practised regularly to form the habits of mind necessary [44, 51, 52]. It has been reiterated by both Ennis [52] and Gelder [51], among others, that practice in varied contexts and manners is important to develop the transferable aspect of the skill; this is needed to achieve the overall aim of increased scientific literacy [53]. For biochemists, this could mean that thought should be given to the development of critical thinking skills in the laboratory environment due to the requirement for critical analysis of experimental design and experimental data, as well as problem‐solving – a distinct but associated higher‐order thinking skill [44]. Problem‐solving in a laboratory environment is essential in order to improve laboratory experiments experiencing technical issues [54, 55]. Therefore, due to the utilisation of laboratory experiments in furthering knowledge and understanding in both learning and research environments, problem‐solving is a key element of biochemical literacy [56, 57, 58].

### Information literacy: a foundation for lifelong learning

**Fig. 4:**
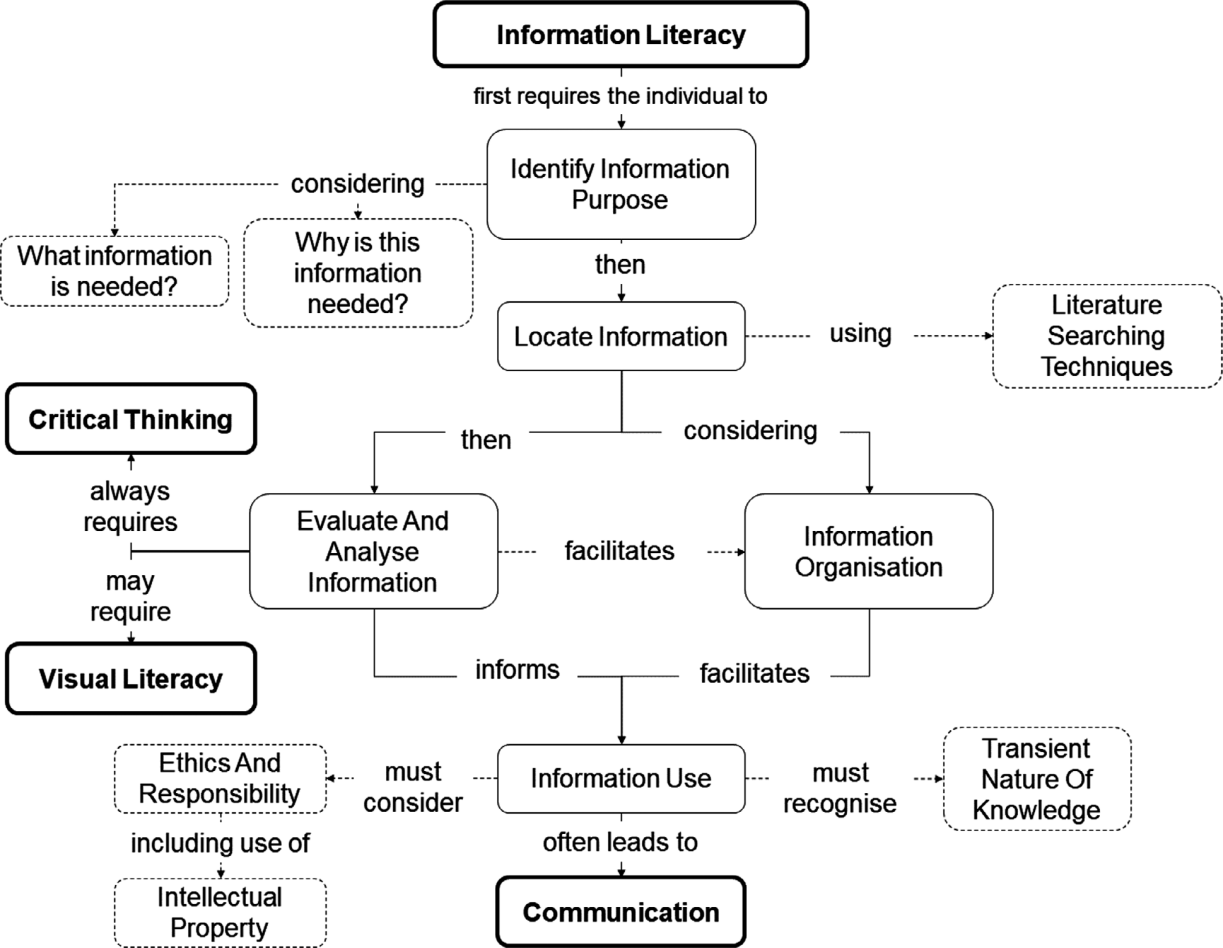
Information literacy. Concept map illustrating the subcomponents of information literacy, linking to other key skills of the biochemically literate individual.

Graduates in biochemistry are encouraged to come to evidence‐based conclusions and thus must be able to use sources of information effectively to inform their decision. This does not mean that every biochemist will come to the same conclusion; however, they should be able to defend their position whilst recognising the transient nature of knowledge, using evidence gathered through thorough critical analysis of information assembled efficiently from a variety of relevant and robust sources. Facilitating this process is information literacy, the subcomponents (including interactions) of which are illustrated in Fig. 4 as a concept map and discussed below [43, 50, 59, 60, 61, 62].

Individuals in any discipline benefit greatly from drawing upon established knowledge to inform their actions both at work and in their personal lives [44, 48, 63]. Drawing upon critical thinking skills, identifying what and why information is needed is often the first step in any project, and is the first step in Fig. 4 [61, 64].

#### Locating information

Being able to locate information competently is essential to allow individuals to inform and expand their learning. A modern graduate must be able to confidently use the tools available to them (linking with technology skills) to learn from a wide range of materials [43, 62].

A literature search begins the information retrieval process, employing a variety of techniques discussed elsewhere [65, 66], and informs the directions and/or methods for research projects. Outside of academia, literature searching is a key skill which when appropriately and robustly applied can streamline the process of finding the most relevant and accurate information to the topic of interest.

#### Analysis, evaluation and organisation of information

As illustrated in Fig. 4, both information organisation and evaluation/analysis occur both concurrently, and in sequence. Continual critical evaluation of information allows robust conclusions to be formed and is vital in both academia and real‐world situations [39, 43]. During a literature search, the individual will continually evaluate the information for relevance and reliability, whilst organising the pertinent information and tracking key concepts. Information can be organised utilising reference management software, drawing upon technology skills and self‐management. This continual evaluation and analysis of information facilitates the creation of connections between ideas, leading to greater understanding – again drawing on critical thinking.

#### Visual literacy

Information can come in many forms, and sources are not restricted to written information. Interpreting information communicated in external representations such as graphs and infographics draws upon ‘visual literacy’. As in information literacy, a critical approach to interpreting external representations of information facilitates building a meaningful understanding of knowledge [67].

Offerdahl *et al*. [68] argued that visual literacy can be considered especially important to biochemists due to the high volume of visual external representations used in the discipline due to the highly complicated systems revealed through modern methods [28, 67]. They additionally make the case that by explicitly including teaching based around developing visual literacy skills, students are more readily able to use nonwritten sources of information to develop and communicate their understanding of biochemical knowledge – becoming more fluent in discipline‐specific discourse and by extension, more biochemically literate [68]. The relationships between molecular form and function are key to understanding content for biochemists [69]. Thus, visual literacy development must not be restricted solely to interpretation and creation of external representations, but also to the individuals innate understanding of a 3D world. It is on this basis that visual literacy has been explicitly included within the BCLF.

#### Intellectual property and information use

During use of information, an understanding and avoidance of plagiarism through citation and awareness of intellectual property is vital. Plagiarism at graduate level is a contentious issue that often makes the national news. In addition, only 40% of UK higher and further education students surveyed by the National Union of Students (NUS) in 2012 considered their knowledge of intellectual property to be sufficient to support them in their future career [70].

### Communication

In the modern world, with its technological advances and cross‐disciplinary work, good communication and collaboration skills are essential. Good communication facilitates change, through collaborations borne out of mutual understanding, and thus is applicable whilst learning, throughout a career and in life. All of the biochemical course guidance places an emphasis on these skills, as critical for the graduate biochemist – regardless of their postgraduate choices [27, 71].

Communication encompasses a wide range of skills, including the use of language (such as appropriate use of discipline‐specific nomenclature), listening skills [72] and using an appropriate format – whether that is written, oral or visual. Science communication facilitates understanding, enabling informed decision‐making [73]. This is particularly important with the use of social media breaking down access to and engagement with science [74, 75], and politics becoming more interwoven through policies based upon and affecting research [76, 77]. Therefore, the communication of scientific information in a format and language appropriate to the audience is a skill that can arguably benefit society as a whole [78, 79, 80, 81].

### Self‐management

**Fig. 5:**
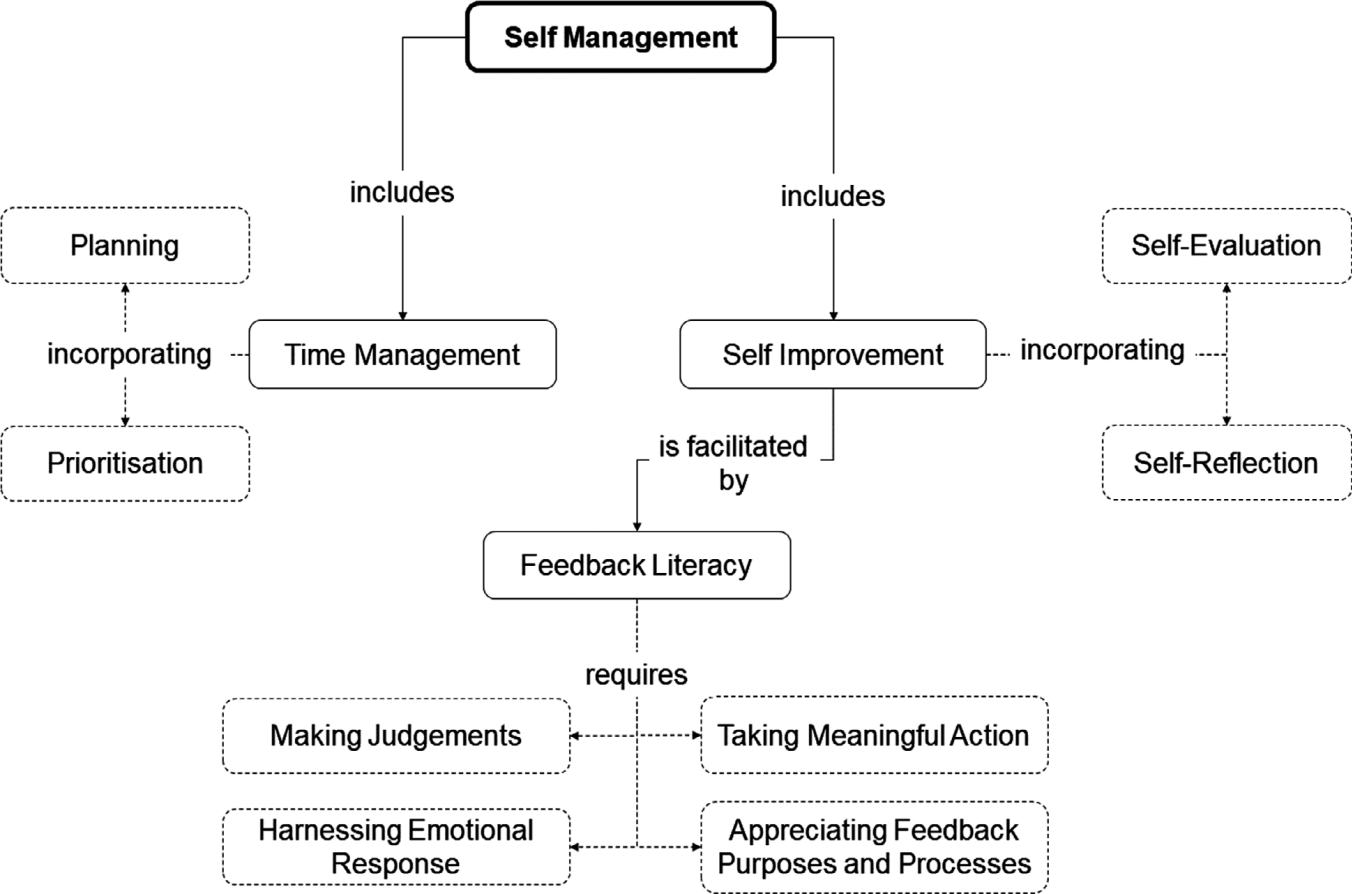
Self‐management concept map illustrating elements of self‐management, including those which support self‐improvement.

There are several skills underpinning self‐management as illustrated in Fig. 5 as a concept map. Self‐management along with autonomy and self‐discipline constitutes three desirable employee characteristics [82]. Self‐management is referred to repeatedly in the QAA, RSB and Biochemical Society guidance – from ‘self‐learning’ and ‘project management’ in the RSB Accreditation documentation [1], to ‘independent learning skills’, the ability to ‘identify and work towards targets’ and being able to ‘evaluate their own performance’ in the QAA Subject Benchmark Statement for Biosciences [27].

Effective self‐management may help in avoiding stress by controlling and directing aspects of learning, as in project management, drawing upon time management skills such as prioritisation and planning. Without organisational and self‐management skills, an individual may struggle to engage fully in the self‐directed learning critical to remaining up‐to‐date in a discipline [83, 84].

Individuals with good self‐management skills may be considered more proficient in their laboratory skills where timing and organisation is essential. These skills are often necessary to complete a biochemistry course with their assessment deadlines and laboratory skill elements, and however, not all students will enter at the same level; therefore, self‐management skills must also be explicitly developed [82, 83].

Self‐improvement is also a factor of self‐management, and as shown in Fig. 5, which relies upon and is facilitated by feedback literacy. Whilst students most likely will have received feedback prior to university, students may begin their biochemistry programmes without having been supported to use feedback effectively [85, 86].

Research suggests that directly training students to manage and use feedback productively can lead to an increase in students' self‐reported feedback literacy [87]. Feedback literacy may not seem to be at the forefront of biochemical priorities when teaching; however, Quinton and Smallbone [88] argue that for ‘learning from feedback to be most effective, programmes should be designed to include classroom time allocated for reflection on written feedback’. The feedback given in such sessions should be timely and accurate [44, 51], in addition to both easy to understand, and highly applicable in order to optimise learning outcomes [87, 89]. Winstone and Millward [90], and Shute [91] discuss the importance of formative feedback, noting several of the advantages as: providing future modification and development guidance; affecting student motivation by highlighting the gap between where the student is, and where the student needs to be; and fulfilling the student desire for feedback which supports a deeper understanding of the subject.

There are three key elements to appreciating feedback purpose and processes and thus increasing feedback literacy: [87, 92]

- Making judgements about the quality of work
- Managing emotion in response to feedback
- Taking meaningful action on feedback

All the elements of feedback literacy should be considered when aiming to facilitate biochemical literacy as without the capacity to identify actions to improve, students may stagnate in their learning.

### Practical skills

There are many practical elements underpinning the biochemical sciences [27]. These are discussed below in detail, but generally support the deeper and more interconnected understanding of biochemical content knowledge.

#### Laboratory skills

The biochemical graduate benefits from good laboratory skills – these skills underpin drug discovery, diagnostic services and the development of consumer goods (e.g. cosmetics, functional foods and cleaning supplies) [93]. Laboratory skills have many elements, which may be best represented in the three domains proposed by Zaghloul [94]: the cognitive domain, the affective domain and the psychomotor domain. The cognitive domain (i.e. how the student's cognitive activities are structured as based on Bloom's taxonomy [95, 96]) links with biochemical content knowledge, in addition to several of the other skills discussed under ‘practical skills’ such as data management, health and safety, and research methods and methodology. The affective domain encompasses the student attitude towards the content knowledge, their education and the laboratory activities – these again link strongly health and safety, in addition to equipment handling. Good laboratory skills must include a grounding in using basic equipment (including but not limited to: a pipette, a microscope, the centrifuge and a spectrophotometer) and following common procedures (including but not limited to: cell culture, aseptic technique, chromatography and electrophoresis) [27]. Finally, facilitating the development of the psychomotor domain, that is, the coordination between the students' brain and body [94], is essential to supporting accurate and precise laboratory practices.

The exact laboratory skills a biochemical graduate should have at their disposal are likely to change frequently with advances in analytical techniques. However, the literate graduate would be confident enough in their laboratory practices to adapt, with appropriate training, to new methods and equipment. These laboratory skills are likely to draw upon technological skills, psychomotor skills, creativity and critical thinking skills in order to support the most adaptable and versatile graduates.

#### Technology skills

The capability for adaption is key in this technologically fast moving era [97]. Adaption to new and emerging technologies, new opportunities and existing technology to new applications are all commonly expected of individuals – both in and out of the workplace [97, 98, 99, 100]. Therefore, graduates in all disciplines must be able to confidently and competently use technology – in particular, to assist in processes such as data management and analysis [101]. Explaining computational ideas in the context of biology could be taught authentically utilising bioinformatics methodology as the context; bioinformatics is an important skill utilised by the modern biochemist, and it is recommended to be taught at the undergraduate level [102, 103, 104, 105, 106].

Online information seeking has been shown to be without depth in the ‘Google Generation’ (born post‐1993), and therefore, appropriate levels of technological skills should not be assumed in undergraduates – these are skills that can be, and need to be taught [43, 107]. Technological skills can be developed alongside other attributes as integrating skills into meaningful tasks is key to disciplinary literacy [108]. For example, statistical analysis of laboratory results to disseminate in a conference‐style poster would draw upon critical thinking, creativity, communication (including presentation), numeracy and technology use.

We have focused little on defining exact technology skills required for the biochemical graduate – this is because, like biochemical literacy, technological literacy is underpinned by cognitive skills we earlier grouped under ‘critical thinking’ [109]. This further supports the notion that key skills should be the focus of educational courses, with subject‐specific knowledge simply the context in which these skills are learnt.

#### Research methods and methodology

An understanding of research methods and methodology facilitates the understanding of scientific processes by enabling the development of connections as well as supporting the logical analysis of information [5, 110, 111]. A greater scientific literacy can be attained by exposing learners to the language common in research – useful to both future ‘users’ and ‘consumers’ of research [110, 111]. Teaching research methods and methodology are more than just exposure to biochemical discourse; it also aims to build a critical approach to attaining and testing knowledge essential to the successful biochemical graduate. Proposing and testing hypotheses is also applicable beyond academic life, allowing the construction and understanding of new knowledge facilitating informed decision‐making [5, 111]. During the design and undertaking of experiments, consideration must be given to validity, accuracy, calibration, precision, duplicability, appropriate use of controls and possible sources of uncertainty or bias.

When testing a hypothesis, consideration must be given to ethics at all levels. Ethics promote truth and minimise error – for example by prohibiting fabrication, falsification or misrepresentation of data [112, 113]. Ethics also protect intellectual property interests whilst still encouraging collaboration by promoting ‘trust, accountability, mutual respect and fairness’ [112]. National and international laws on the conduct of research (particularly involving animal or human subjects) help to promote and enforce an ethical research environment [112, 114]. Due to all of these reasons, and more, ethics must be embedded into the teaching of research and research methodology, and biochemistry.

#### Data management

Linking with practical laboratory skills are data management skills. Data management is required on several scales as a biochemist – from the physical organisation of stored samples, and the maintenance of a laboratory book to large data sets and meta data. The appropriate transformation, analysis and interpretation of experimental data using either/both qualitative and quantitative techniques involve the use of good numeracy skills [27, 115].

Numeracy skills are vital for on‐the‐go calculations in the laboratory environment; therefore, these skills must also be taught and reinforced. To facilitate analysis of data, the use of statistical programmes and spreadsheets is beneficial – again linking with technological skills.

#### Health and safety

Grouped under research methods and methodology are the health and safety considerations that must underpin testing and laboratory skills. These are vital for the undergraduate biochemist to participate in laboratory‐based learning tasks, as well as for the graduate biochemist in a research or specimen analysis role. The fundamental idea of ‘risk assessment and minimising risk’ learnt in the laboratory applies widely in workplaces across industry sectors, with consideration given not just to one’s own health and safety, but the health and safety of others too. A variety of teaching methods can be used to educate in health and safety [116, 117], but it is a vital aspect of all undergraduate biochemistry courses, and thus is included in the BCLF. The topics that may be covered under health and safety include Control of Substances Hazardous to Health assessments and the use of Personal Protective Equipment.

### Content knowledge – a conceptual perspective

Biological knowledge is complex, progressing alongside scientific advances and giving rise to a dynamic and changing discipline. Much of taught knowledge is conceptual rather than specific, and these concepts are illustrated with examples to enhance perspective and comprehension [28]. To understand the biological sciences is to have an ‘understanding of the processes and mechanisms of life’ [27], from the molecular to the cellular and beyond. Due to the many inter‐relationships inherent in studying the processes and mechanisms of life, the biological sciences are underpinned by chemistry, mathematics and physics in addition to the data analytics and information technology skills previously discussed.

Due to the unique viewpoint biochemists utilise, biochemistry requires a strong foundation in chemistry; chemical principles relate to important biochemical concepts, therefore enabling more complete understanding and deeper study. Not all students enter university with the same grounding in chemistry, thus ensuring the basics are embedded early in courses (though these do not have to be in chemistry‐specific modules/courses [118]) is vital in strengthening and supporting key concepts across a wide range of interacting subdisciplines including physiology, genetics, microbiology and pharmacology.

Biochemistry uses chemical knowledge and techniques to understand and solve biological problems, focusing on biological processes within and related to living organisms. ‘Science is not a body of information to be mastered, but rather a way to construct new knowledge’ [5]. For example, biochemists use their understanding of how structure relates to function in a molecule to predict how that molecule will interact within a biological system. A conceptual approach to knowledge helps to facilitate these cross‐disciplinary interpretations, and there are several ‘key concepts’, which provide a good foundation for cross‐disciplinary learning [119, 120, 121].

#### Identifying key concepts

**Fig. 6:**
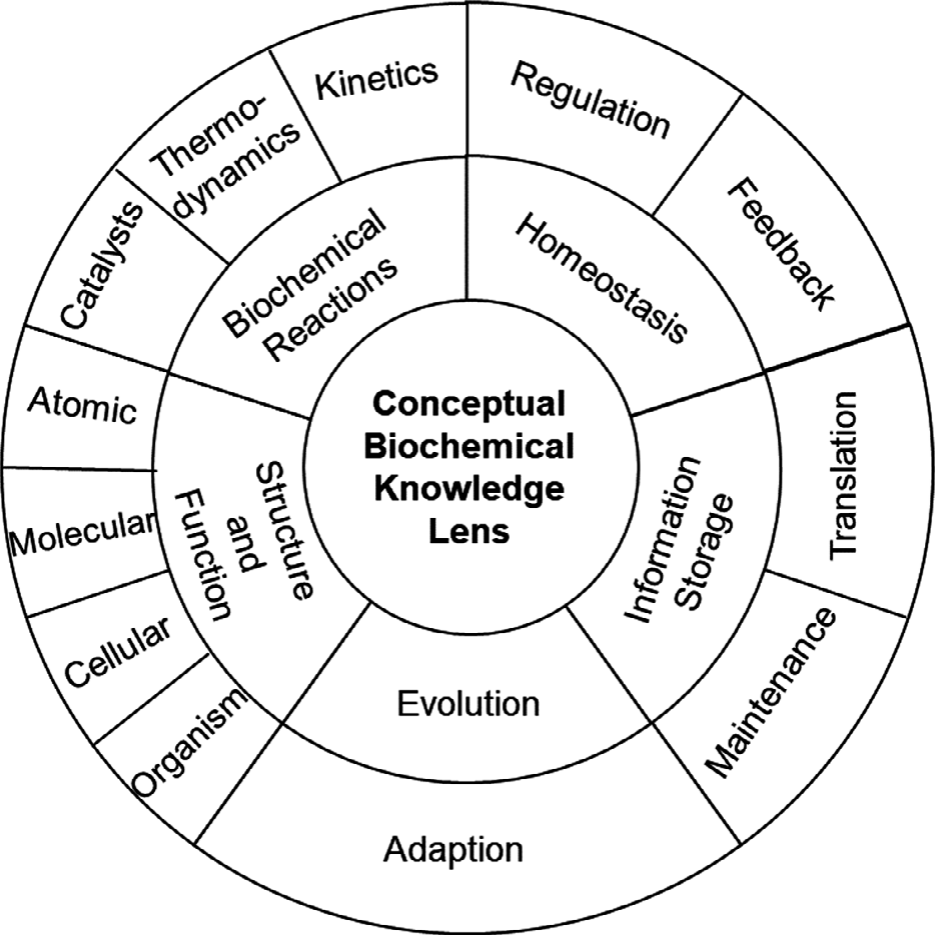
Conceptual biochemical knowledge lens illustrating the five major conceptual ideas of biochemistry and their subcategories around which curriculum design can be discussed. Based upon the research of the American Society of Biochemistry and Molecular Biology [128].

Several key concepts are mentioned in each course content guidance document examined – we have identified and categorised these for clarity during discussions in Fig. 6.

Each of the guidance documents referred to five key concepts – homeostasis, biochemical reactions, structure and function, evolution and information storage. These five key concepts are not categorical and interact with each other – for example, the structure of a molecule determines its function, which may be as a catalyst in a biochemical reaction. The subconcepts were chosen from the guidance to give an indication of the scope of the key concept; however, teaching should highlight the interactions to provide a more comprehensive and authentic understanding of biochemical knowledge [122, 123]. Further exploration of the key concepts of biochemistry may benefit curriculum design and have already begun [120, 124, 125, 126].

## Conclusion

The term ‘scientific literacy’ has been in use for many decades, with several definitions. Scientific literacy is the ability to ‘make use of scientific knowledge in real‐world situations’ [127], that is both not limited to academia and touching upon the importance of scientific literacy in everyday life for every citizen. Holbrook and Rannikimae [55] discuss the varying definitions proposed, and identified the defining concept as ‘Scientific literacy is not simply reliant on the acquisition of content’. This is the underpinning concept of the BCLF and the basis of the representation of skills leading to the understanding of biochemical content knowledge in Fig. 2. It is intended that this framework be used to pragmatically approach the complex nature of curriculum design in the biochemical sciences within higher education institutions.

The seven skills (critical thinking, information literacy, visual literacy, self‐management, communication, practical skills and content knowledge) of the biochemically literate individual proposed here are not intended to be an unchangeable, rigid framework for curriculum design. Their interaction and connections are far more important than the categories in which they have been placed for clarity of communication. This is intended to support the creation of evidence‐based programmes of learning – focusing on the interconnections of biochemical knowledge, fostering lifelong learning skills and developing confident curious open‐minded biochemists. We intend that this proposed framework be utilised as the basis of discussion and innovation when developing biochemical curricula constructed around the idea of ‘skills in the context of disciplinary content knowledge’.

Future work will include the exploration of academic, student and industry definitions of biochemical literacy (overall, and by dimension) with the particular aim to validate the current proposal in line with the other disciplines discussed. Additionally, responses to the proposed BCLF will be invited to explore where further work might be directed.

## Conflict of interest

The authors declare no conflict of interest.

## Author contributions

DLE, IGB, NEW, AET, SGB and SLT involved in the conceptualisation. DLE performed the data curation, resources and investigation, and involved in the project administration and visualisation. IGB, NEW, SGB, SLT and AET involved in the funding acquisition. DLE, IGB, NEW and AET performed the methodology. IGB and NEW involved in the supervision. DLE, IGB, NEW and SGB wrote original draft preparation. DLE, IGB, NEW, SGB and SLT reviewed and edited the manuscript.

## Data Availability

All data used in the creation of this manuscript are available in the public domain.
